# Perceived Drivers and Barriers to the Adoption of eMental Health by Psychologists: The Construction of the Levels of Adoption of eMental Health Model

**DOI:** 10.2196/jmir.9485

**Published:** 2018-04-24

**Authors:** Milou A Feijt, Yvonne AW de Kort, Inge MB Bongers, Wijnand A IJsselsteijn

**Affiliations:** ^1^ Human-Technology Interaction Department of Industrial Engineering & Innovation Sciences Eindhoven University of Technology Eindhoven Netherlands; ^2^ Tranzo Tilburg School of Social and Behavioural Sciences Tilburg University Tilburg Netherlands

**Keywords:** eHealth, mental health, psychology, clinical, diffusion of innovation, technology

## Abstract

**Background:**

The internet offers major opportunities in supporting mental health care, and a variety of technology-mediated mental and behavioral health services have been developed. Yet, despite growing evidence for the effectiveness of these services, their acceptance and use in clinical practice remains low. So far, the current literature still lacks a structured insight into the experienced drivers and barriers to the adoption of electronic mental health (eMental health) from the perspective of clinical psychologists.

**Objective:**

The aim of this study was to gain an in-depth and comprehensive understanding of the drivers and barriers for psychologists in adopting eMental health tools, adding to previous work by also assessing drivers and analyzing relationships among these factors, and subsequently by developing a structured representation of the obtained findings.

**Methods:**

The study adopted a qualitative descriptive approach consisting of in-depth semistructured interviews with clinical psychologists working in the Netherlands (N=12). On the basis of the findings, a model was constructed that was then examined through a communicative validation.

**Results:**

In general, a key driver for psychologists to adopt eMental health is the belief and experience that it can be beneficial to them or their clients. Perceived advantages that are novel to literature include the acceleration of the treatment process, increased intimacy of the therapeutic relationship, and new treatment possibilities due to eMental health. More importantly, a relation was found between the extent to which psychologists have adopted eMental health and the particular drivers and barriers they experience. This differentiation is incorporated in the Levels of Adoption of eMental Health (LAMH) model that was developed during this study to provide a structured representation of the factors that influence the adoption of eMental health.

**Conclusions:**

The study identified both barriers and drivers, several of which are new to the literature and found a relationship between the nature and importance of the various drivers and barriers perceived by psychologists and the extent to which they have adopted eMental health. These findings were structured in a conceptual model to further enhance the current understanding. The LAMH model facilitates further research on the process of adopting eMental health, which will subsequently enable targeted recommendations with respect to technology, training, and clinical practice to ensure that mental health care professionals as well as their clients will benefit optimally from the current (and future) range of available eMental health options.

## Introduction

The internet offers major opportunities in supporting mental health interventions [1]. A variety of technology-mediated mental and behavioral health services are available [2,3], with a growing body of evidence supporting their efficacy (eg, [4-7]). Over the past decades, a mix of terms and definitions has been used to describe eletronic mental health (eMental health; eg, [8-10]). This study will use the term eMental health to refer to “any delivery of mental and behavioral health services, including but not limited to therapy, consultation and psycho-education, by a licensed practitioner to a client in a non-face-to-face setting through distance communication technologies such as the telephone, asynchronous email, synchronous chat, and videoconferencing [8].”

Unique benefits of eMental health tools include increased access to psychological treatment, convenience, as well as enhanced self-reflection and increased emotional disinhibition of the client [9,11,12]. These positive findings, however, are in contrast to the low adoption (ie, acceptance, uptake and use) of eMental health by psychologists. Although exact numbers on a national or international level are scarce, the World Health Organization reported that in 2015 only a third of its member states indicated to have at least one program for technology-mediated mental health services [13]. Moreover, the report shows that most of these programs have a small scale and consist primarily of pilots or informal projects.

Multiple studies have been conducted to investigate therapists’ attitudes toward eMental health, and several possible impeding or facilitating factors for adoption have been identified. A barrier frequently reported by therapists pertains to the lack of the full range of nonverbal cues during mediated communication, as they feel this heightens the risk of misunderstanding and does not allow for the development of a strong therapeutic relationship [14]. It has to be noted though that systematic studies that investigate these concerns are lacking [15]. A major concern often reported by therapists is how to deal with crisis situations online (eg, when a client expresses suicidal thoughts) [16]. Technology-mediated modalities allow clients to disconnect at any time, without the therapist knowing whether this is due to technology failure or because the client is in some kind of crisis, and the therapist is not in the same space to ensure their safety [17]. Another reason found for therapists’ reluctance is the risk of clients misrepresenting themselves, as it is harder to verify an individual’s identity when interacting remotely [17,18]. Therapists also mention more practical concerns such as costs of setting up and maintaining the infrastructure, licensure and jurisdiction constraints, lack of clear ethical guidelines for practice and confidentiality, patient privacy, and the potentially detrimental effects of technology failure [9,14,16,17]. In addition, some studies emphasize the importance of contextual factors of daily clinical practice such as the level of knowledge and training, available time and resources, perceived social norms, forces within the current care system, and the design and usability of the technological tools [14,16,19-25]. These factors vary between different mental health care institutions (eg, whether or not the management of a mental health care institution has allocated time during working hours to invest in eMental health), and in this way the institutional context of a psychologist also has a significant influence.

Despite these efforts to clarify therapists’ perceptions toward eMental health, the exact nature of therapists’ reluctance to its adoption has remained hard to grasp, and as a result, attempts to increase the uptake and use have not been very successful [26]. eMental health comprises a new way of working for psychologists, as they have to integrate new tools into their existing clinical practice. This requires psychologists to change their current behavior and adopt new behaviors. There is a vast body of literature on behavior change and the adoption of innovations, and multiple theories and models have been developed in an attempt to understand or explain influencing factors. Some prominent ones are the diffusion of innovation theory (DIT) [27], the theory of planned behavior [28], the transtheoretical model [29], and the technology acceptance model (TAM) [30]. For extensive reviews of behavior change and implementation theories, models, and frameworks, see Davis et al [31] and Nilsen [32].

Some studies have applied these theories to the topic of eMental health (eg, [14,20,22]), whereas other studies took a more exploratory approach (eg, [21,23,24,33,34]). However, this research has mainly resulted in lists of factors that impact the adoption of eMental health without structuring their relative weights or considering the influence of individual differences in practitioners’ willingness and experience to explore and use technology-mediated therapeutic tools. Moreover, these studies have mostly employed written questionnaires for data collection, which afford a large sample size but restrict in-depth understanding of therapists’ experiences. Another limitation in studies on therapists’ adoption of eMental health pertains to the prevalent focus on barriers within those studies. This probably reflects the relatively large proportion of psychologists who have not adopted eMental health. Thus, a random sample drawn from this population, typical for survey studies, includes only a small percentage of active users and a much larger percentage of nonusers (eg, [22,34-37]). Although nonusers may still see advantages in eMental health, it is fair to say that nonusers will be more focused on barriers to adoption than active users. As yet, relatively little attention has been given to identifying perceived drivers, whereas research shows that perceived value is an important factor in reducing resistance to use a new technology [38], and some studies even suggest that perceived benefits are rated as more important than perceived barriers in the decision to use a novel technology [39,40]. Moreover, research shows that perceiving a new technology as advantageous compared with the existing practices is a key factor in the adoption of an innovation [27]. Hence, this research employed a stratified sampling strategy that specifically allows for *both* potential drivers as well as barriers in adopting eMental health to emerge.

In addition to its emphasis on barriers, the current literature tends to present both drivers and barriers as relatively undifferentiated lists of factors. How these factors combine or relate to each other and to the level of an individual’s acceptance and use of technology-mediated therapeutic tools has been relatively unstudied. A conceptual model describing the interrelationship between these factors could lead to a more structured understanding of the process of technology acceptance and use in relation to eMental health tools in psychologists’ clinical practice. In turn, these structured insights may help address and prioritize selected drivers and barriers over others, thus potentially informing processes of technology development, interface design and evaluation, professional training and coaching, targeted clinical use, and organizational embedding of eMental health tools.

The aim of this study, therefore, was to gain an in-depth and comprehensive understanding of clinical psychologists’ perspectives on the adoption of eMental health tools. To reach this objective, the authors will identify both drivers and barriers and analyze possible relationships among the involved factors and strive to structure the obtained findings. This will subsequently enable targeted recommendations with respect to technology, training, and clinical practice to ensure that mental health care professionals as well as their clients will benefit optimally from the current (and future) range of available eMental health options in mental health care.

## Methods

### Design

This study adopted a qualitative descriptive approach consisting of in-depth semistructured interviews with clinical psychologists. The study consisted of 3 phases. First, a qualitative data collection and analysis phase was aimed at gathering in-depth information about the drivers and barriers to adoption of eMental health from the perspective of clinical psychologists. Second, based on these qualitative findings, a model was constructed that captures different levels of adoption of eMental health and the drivers and barriers related to each level. Third, the model was validated through the process of communicative validation, that is, a second round of interviews to examine whether the model matched the perceptions and experiences of the participants.

### Sampling and Recruitment

The sample consisted of practicing clinical psychologists working in the Netherlands. A total of 17 individuals were approached via emails through referrals from contacts in the health community, of which 12 agreed to participate in the first part of the study and 11 also participated in the communicative validation. Ethical approval was granted by the Eindhoven University of Technology Research Ethics Committee (ID: 581), and each participant was offered a €12 gift as a small token of acknowledgment.

The strategy of theoretical sampling was used—a process in which data are simultaneously collected and analyzed to determine who to approach next to yield (most) new insights [41]. The eventual sample size was determined by a saturation criterion, which is generally defined as the point where no new themes, findings, concepts, or problems emerged from the data [42]. This procedure involves the specification of a minimum sample size for initial analysis and a stopping criterion, that is, how many more interviews will be conducted without new information emerging before it is concluded that the point of saturation has been reached. On the basis of earlier findings, this study employed an initial sample size of 10 participants, and a stopping criterion of 2 participants [43]. As no new themes emerged during the last interviews, the data collection was terminated after interviewing 12 participants. In contrast to earlier questionnaire studies, which mostly used convenience samples, this study used stratified sampling to ensure the inclusion of clinical psychologists with different levels of use and experience with eMental health, and to represent a mix of age, gender, job position, and type of mental health care institution. Table 1 shows the distribution of these characteristics.

### Procedure of the Interviews

Before the start of the interviews, participants were informed about the purpose and content of the study and signed an informed consent form. The interviewer followed a semistructured interview guide containing a mix of both open-ended and closed questions. The interview guide consisted of 29 questions. The topics covered via these questions were based on findings from previous research described earlier [9,11,12,14-16,18-25] and pertained to participants’ knowledge, experience and attitudes toward the use of eMental health tools, covering current use, perceived advantages and disadvantages, and influences of their working environment. Each interview lasted between 45 and 60 min. Most interviews were held at the offices of the participating clinical psychologists or otherwise in a quiet public space. If preferred by the participant, the interview was conducted via telephone or video call (2 interviews). With participants’ consent, interviews were audio-recorded to allow for transcription and subsequent analysis. All files were stored in a secured location accessible only to the interviewer.

In the service of the communicative validation, a second interview was scheduled with the same participants (N=11). Only one participant could not participate in this second round due to restricted availability during the time period of the study. However, because of the diversity of the sample’s characteristics, the authors believe that this has not compromised the validity of the results. At the start, all participants were given a print of the constructed model, a short summary of the results, and a document with statements about the different levels that characterized them. The interviewer followed a semistructured interview guide with questions focusing on the general impression of the model and whether it matched their perceptions and experiences. These interviews lasted between 20 and 40 min.

**Table 1:** Demographic details of the participants (n=12).

Characteristic		n (%)
**Age (years)**		
	20-30	2 (17)
	31-40	3 (25)
	41-50	3 (25)
	51-60	3 (25)
	60+	1 (8)
**Gender**		
	Male	4 (33)
	Female	8 (67)
**Mental health care setting**		
	Public	6 (50)
	Private institution	3 (25)
	Private practice	3 (25)
**Level of use**		
	No use	2 (17)
	Minimal use	2 (17)
	Passive use	2 (17)
	Active use	4 (33)
	Innovative use	2 (17)

### Analysis

The interviews were transcribed and analyzed using QSR International’s NVivo 11 software. The researchers employed a thematic analysis approach to derive themes in participants’ perceptions of barriers and drivers to accept eMental health. The transcripts were systematically analyzed using the procedure outlined by Boeije [44], consisting of 3 phases: open coding, axial coding, and selective coding, resulting in a list of codes, categories, and main themes. Commonly used indicators for the quality of qualitative research are internal validity or credibility, reliability/dependability, objectivity/confirmability, and external validity/transferability [45]. This study addressed these criteria by applying strategies described by Miles and Huberman [45] and Wester and Peters [46]. Credibility was ensured by communicative validation. Dependability was established by performing a coding check on part of the data by an independent scholar. The interrater reliability was determined at a Cohen kappa of .78, which is considered substantial [47] and acceptable for exploratory research [48]. The dependability criterion was further supported by peer debriefing, which consisted of discussing findings with peers and colleagues on various moments during the process. This also enhanced confirmability, as was providing clear examples of key themes. Finally, to improve transferability, theoretical sampling was used with the inclusion of psychologists in various job positions, mental health care institutions, as well as age and level of adoption of eMental health. In addition, connecting the results to previous theories further helps in establishing this criterion.

## Results

### Qualitative Data Collection and Analysis

The results of the interviews are structured along 4 main themes that emerged from the thematic analysis: general characteristics of eMental health, drivers for the adoption of eMental health, barriers for the adoption of eMental health, and contextual factors of daily clinical practice. Within these themes, several subthemes were identified that provide more detailed information.

#### General Characteristics of eMental Health

All participants clearly expressed the indispensability of face-to-face contact for the delivery of their treatment, because they felt mediated forms of communication lack the subtle signs in facial expressions, posture, and appearance they believe to be crucial for an accurate understanding of their client. In line with this, all participants stated they only wanted to use eMental health in combination with face-to-face sessions, as a complement to their treatment. One participant said:

I do not think it can truly be a replacement. Because the way that people behave and look is indispensable… I particularly find it a very nice complement to my treatment.

P3

Another general point emerging from all the interviews is that not every kind of eMental health works for every client; it strongly depends on the client’s specific needs, capabilities, and preferences, which is clear from the following statement by one of the participants:

You have to make eHealth really adapted, customized; what does that client need, and what fits the specific situation.

P3

Factors often mentioned to have an important influence on the specific utilization of eHealth were the nature and complexity of the psychological disorder, the client’s age, level of computer skills, intelligence, and the devices available to the client. This was evident by what one of the participants expressed:

Not everyone is equally skilled with computers, that does make a difference too. Then just using the discussion feature [of the eMental health platform] is already an accomplishment.

P7

Besides characteristics of clients, participants also reported characteristics of therapists that made them more or less suitable such as the level of computer skills, affinity with technology, age, and therapeutic approach:

But I have to admit, I do not even use...I am very bad with computers, so I do not have any experience with all those online telephone things. So I don’t know how that would feel in my day-to-day work.

P8

#### Perceived Drivers to the Adoption of eMental Health

All participants agreed that one needs to be convinced of the benefits to adopt eMental health:

If I would know that it would bring something positive to my clients, then I would definitely be much more motivated.

P8

Furthermore, it became clear that beyond being or becoming aware of the benefits, experiencing them is crucial in decreasing resistance and developing intrinsic motivation to continue using eMental health. Reiterating this, one of the participants said:

I become more and more aware of the benefits, definitely. Especially with my target group, addiction, I experience that it truly is an addition [to the treatment].

P1

A frequently mentioned benefit was that mediated contact in-between regular sessions affords a more intimate and personal therapeutic relationship, because it increases the frequency of contact between the therapist and client, and in this way enhances a sense of continuity:

People are making a stronger link with you, like “hey, you also think about me outside of that room.” And that is very beneficial to your relationship with people, they really feel I still exist for them.

P6

Most participants using eMental health reported that this increased frequency of contact intensifies the treatment. Moreover, it stimulates clients to engage in a higher level of therapeutic activity at home, thus accelerating the therapeutic process:

eHealth gives you something of an intensifier. People can work on something every day.

P11

Several therapists recognized the benefit that both the technology-mediated interactions between sessions and heightened client activity at home allow for more consolidated progress. In addition, the higher frequency of interactions allows for the introduction of new therapeutic elements during the actual face-to-face sessions, rather than the repetition of earlier steps in the process. In the words of one participant:

I notice that they really are much more active at home and also return to the next session with more to discuss. Or that they have already thought about that, whereas with others you have to make them reflect at that moment itself, and then it is hard sometimes, then time passes by much quicker and you can make less progress, whereas when you make them work at home then you find that you can do much more in the sessions too.

P9

Moreover, in some cases eMental health allows for a better satisfaction of client needs, for example, when it is very burdensome for clients to travel to the therapist’s office, when they are abroad, or in cases of illness, pregnancy, or other limiting circumstances. One participant stated:

I regularly have videoconferences with people with young children who cannot leave their house, or who are too ill to come. I also had several people living abroad, then it is also pretty convenient.

P3

Although less prominent, several therapists mentioned practical personal benefits of eMental health, such as increased efficiency in administrative tasks. Finally, some expressed that their enthusiasm toward eHealth was mostly due to the new treatment possibilities offered by eMental health, such as interventions with virtual reality and biofeedback, enabling them to treat their clients in ways that were not possible before.

#### Perceived Barriers to the Adoption of eMental Health

Perhaps the most important barrier reported by participants was a lack of knowledge and experience on their side with respect to various aspects of eMental health such as how to integrate eHealth into their treatments and the possibilities of available tools. This last point seems to be partly due to the relatively large—and fast growing—number of available tools, combined with the lack of a comprehensive overview of the availability, relevance, and efficacy of technology-mediated therapeutic tools in their clinical context. This was reflected in the statement made by one participant:

Sometimes I hear something from a colleague that I say “oh, is that available? I did not know.” And if I am not aware of something, then I will not use it.

P7

Related to this, an important barrier was the strong professional obligation most participants feel to be an expert in eMental health before they can apply it in their daily practice, as is evident from the following statement:

I feel like it does not come across professionally to set up such an eHealth module with someone if I would not know exactly how it works. I find that unacceptable.

P12

Several participants reported that it would be helpful if they would receive training and have the opportunity to practice more with an eMental health platform and try out various tools:

It would make a big difference if I could practice more with the system. Because then it becomes familiar, whereas now it is not.

P12

As a counterpoint to the advantages of increased availability through eMental health tools described above, participants mentioned that being more accessible to clients also comes at a cost. It may put a higher demand on therapists by increasing the communication channels they must keep track of and the times at which they have to make themselves available to their clients. This is echoed by the following statements by 2 participants:

Now I not only have to watch my email, not only my phone, but then I also have to watch that Whatsapp.

P10

In a way eHealth makes the job more burdensome. You have to juggle multiple tasks as a mental health professional.

P4

Increased accessibility sometimes also increases feelings of responsibility, especially when dealing with crisis situations. This issue seems to be complicated by the lack of clear ethical guidelines and instructions on how to handle these kinds of situations:

If I am home at night and I read the message that someone is suicidal, what do I do? So that aggravates matters, because you feel like you always have to be available and you also wonder “where do my responsibilities lie?"

P6

Both increased availability and sense of moral responsibility beyond office hours are likely to add to the stress and workload that therapists experience and may negatively affect the balance between a therapist’s work and private life.

Furthermore, all participants who were using eMental health tools experienced some technological issues. Most frequently mentioned were usability problems such as having troubles with logging into the system, and functionality issues of the eMental health platform, for example, the lack of an adequate search tool to explore the available content. In addition, complaints with respect to the quality of the videoconferencing technology were reported frequently.

#### Contextual Factors of Daily Clinical Practice

An experience that was reported in every interview was a perceived pressure from managers and health insurance companies to use eMental health. Some participants expressed not to be bothered much by this, but for others this caused a general sense of distrust against management, feeling that their management’s interest in eMental health was solely driven by the goal to save money and not because psychologists or their clients would benefit from it. This elicited strong feelings of resistance. As one of the participants stated:

That is how eHealth is often looked at, like it is just a way to claim a two percent or higher hourly rate from the insurance companies.

P4

Another common experience was a high pressure on productivity during work and a lack of organizational support in terms of time and resources. Participants argued that not having sufficient time to invest in eHealth during working hours significantly hinders the implementation of eMental health:

There is nothing that exploring [eHealth] can be registered as, so then it reduces my productivity rate and then I think “no, I will not do it. That will only cost me and does not benefit me at all.”

P12

Several participants also mentioned the low visibility and awareness of eMental health during daily practice as a prominent obstacle to the adoption of eMental health. Some attributed this to the topic of eMental health not being discussed much among colleagues or in team meetings:

I just barely encounter it, so then it also fades to the background more easily.

P9

eHealth is primarily a topic that is just not that much discussed.

P8

As a solution, participants suggested that external triggers and standard procedures such as automatic reminders would be helpful to facilitate the adoption of eMental health. In addition, they mentioned the importance of keeping an open dialogue on the topic to trigger awareness and reflection upon one’s opinion about eMental health.

### Construction of the Levels of Adoption of eMental Health Model

The previous sections described sets of drivers and barriers, as well as contextual conditions that affect the adoption of eMental health tools. An important insight that emerged across the various interviews is that although some experiences and attitudes are common among the entire sample, a strong differentiation could be seen in the nature and importance of the various drivers and barriers perceived across the participant sample; that is, the list of drivers and barriers was not uniformly or randomly distributed across the interviewees but rather seemed to be related quite strongly to the actual exposure and hands-on experiences that people had had regarding eMental health tools. This is in line with the notion, discussed in the Introduction, that the adoption of eMental health tools, similar to the adoption of any technological innovation, requires psychologists to adopt new behaviors.

A model that seems to be particularly well suited to describe the process of behavior change to adopt innovative technology is Rogers’ well-established diffusion of innovations theory (DIT) [27]. The DIT states that people differ in the time they need for adoption because of several individual characteristics and Rogers has distinguished 5 discrete adopter categories from fast to slow adoption based on these differences. This conception seems to be applicable to this study, as differences were found within the group of participants in the extent to which they had adopted eMental health. On the basis of similarities between the characteristics of Rogers’ adopter categories and characteristics of the participants regarding their attitude and use of eHealth tools, the authors determined 5 levels of adoption of eMental health (see Table 2).

**Table 2:** Adopter categories from Rogers’ diffusion of innovations theory (DIT) and their corresponding levels of adoption of electronic mental health (eMental health).

Adopter category by Rogers	Level of adoption of eMental health
Laggards	No use
Late majority	Minimal use
Early majority	Passive use
Early adopters	Active use
Innovators	Pioneer or innovative use

**Figure 1:**
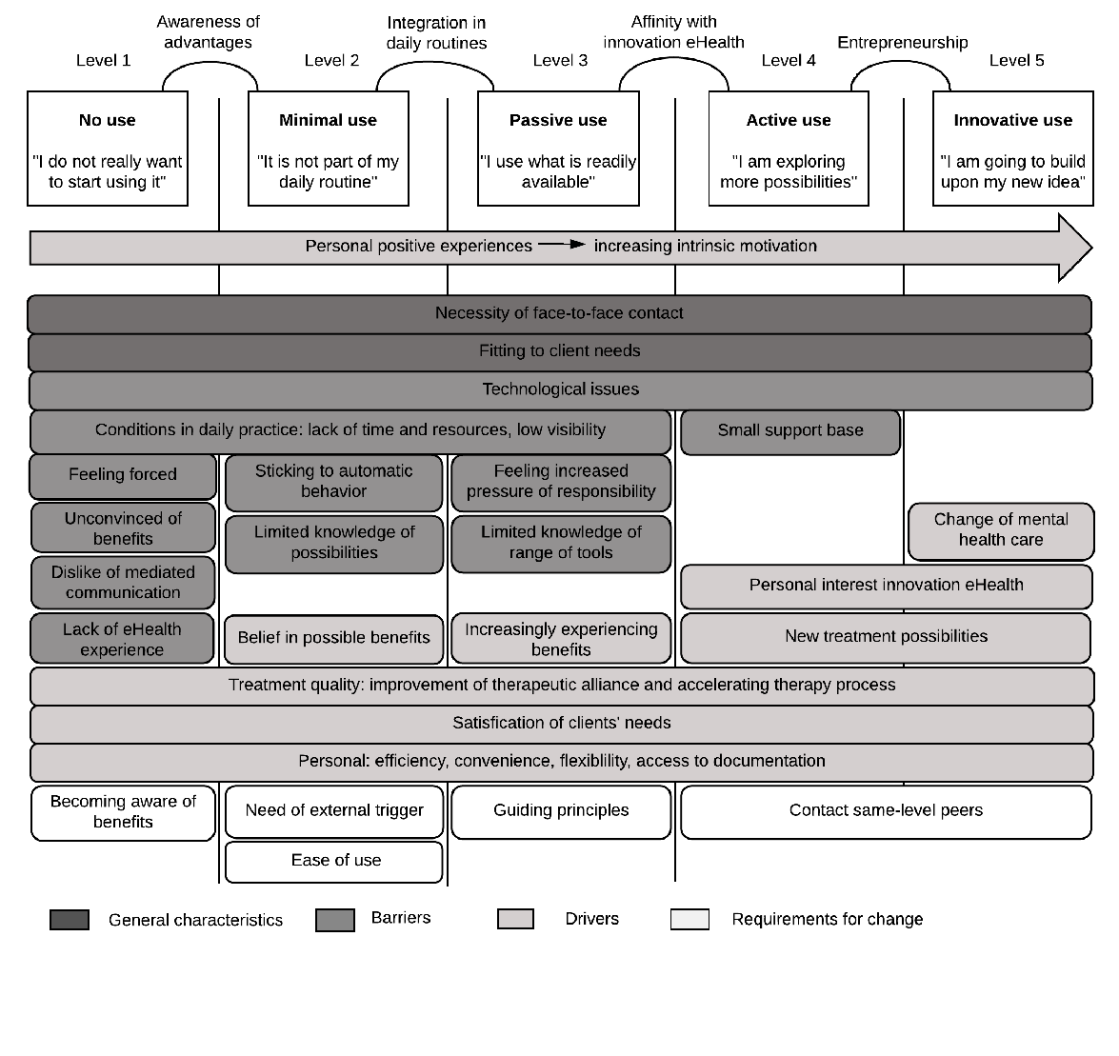
The Levels of Adoption of eMental Health (LAMH) model.

When applying these levels of adoption—that is, the extent to which clinical psychologists had already adopted eMental health in their clinical practice—clear differences can be found in the types of perceived drivers and barriers that characterize the psychologists at the different levels of adoption, as well as differences in the types of processes required for psychologists to move from one level of adoption to the next. Developing this notion, the authors propose the Levels of Adoption of eMental Health (LAMH) model. This model incorporates the 5 levels of adoption identified by the authors and links them to the general characteristics, drivers, barriers, and requirements for change that were found relevant for each level. These factors are directly derived from the main themes extracted during the thematic analysis of the in-depth interviews and hence are entirely based on the perceptions expressed by the participants. The final LAMH model is shown in Figure 1.

The 5 top rectangles in Figure 1 present the 5 levels of adoption, including a characterizing phrase that the authors formulated based on the interviews, exemplifying the attitude of a typical user at each level. The text above the connecting lines between levels describes the factor that was found to distinguish these levels. The bars below the rectangles represent factors that constitute general characteristics, drivers, barriers, and requirements for change. When a factor is located under a particular level, this means that it is most important for clinical psychologists at that level.

Clinical psychologists at level 1 (No use) are generally not convinced and even skeptical about the advantages that eMental health can have and are therefore averse to using it. When they experience pressure by management to do this, it results in a strong feeling of resistance. Psychologists in this group might also feel that eMental health does not suit their profession and show an aversion for computer-mediated communication in general. Psychologists in this category are further characterized by a relatively low level of computer literacy and lack of exposure to eMental health. In accordance with this, use of eMental health tools is nearly or entirely absent, possibly with the exception of telephone, and email for administrative purposes (ie, to schedule an appointment).

At level 2 (Minimal use), clinical psychologists are becoming convinced that eMental health may carry some advantages, which is the most important distinction between levels 1 and 2. However, they are generally unsure how to implement it into their daily practice. Because their intrinsic motivation is fairly low, participants do not want to spend a lot of time and effort in learning to use eMental health. Therefore, there tends to be a lack of knowledge about the possible ways in which eHealth can be applied in practice, and the use of eMental health is likely to be restricted to familiar and easy-to-access tools such as email, telephone, and instant messaging. Functionality limitations and usability issues have a relatively large influence in this group and ease of use of the eMental health tools is a major requirement. Because applying eMental health tools is not integrated into their daily practice, psychologists at this level are prone to maintain their existing way of working and as a result do not gain the positive experiences that could increase their intrinsic motivation.

Clinical psychologists at the third level (Passive use) are using eMental health tools as part of their daily routines. In a positive situation, this daily use leads to an increased conviction of the added value of eMental health, as they gather more and more positive experiences. However, regular use also might confront them with the challenges and limitations of eMental health such as the perceived lack of nonverbal communication or concerns about the pressures and responsibilities associated with being much more easily accessible. Although members of this group are generally motivated to use eMental health, they tend to stick to the applications that are readily available and are not inclined to actively search for other possibilities, which results in a limited overview of the entire range of eMental health tools and limited in-depth knowledge of specific tools. Their use of tools mostly consists of familiar applications such as email, telephone, and WhatsApp, or other mobile phone apps that are relatively well-known, for example consisting of mindfulness exercises. When easily available, an eHealth platform is regularly used.

Compared with practitioners at level 3, clinical psychologists at level 4 show a high level of personal interest in the developments of eMental health and hence have a higher intrinsic motivation to actively keep track of new developments in the field of eMental health. The new treatment possibilities technology-mediated tools offer can act as an additional driver for this group. They make use of a broad range of tools and are eager to try newly available options such as virtual environments or digital games. Most psychologists in this group function as experts of eHealth within their working environment and might be one of the few in their team actively using eMental health. The lack of interest from colleagues may lead to frustration, and frequent contact with same-level peers is important to support their positive attitude toward eMental health.

The characteristics of level 5 are largely similar to those of level 4. The most pronounced difference can be described as entrepreneurship, that is, the initiation of projects to develop and test new eMental health tools or apply the existing tools in novel contexts. In addition, the participants in the highest level might also have a clear vision about major and largely positive changes they expect eMental health will bring to the field of mental health care and even society in general, such as the enabling of personalized care and the opportunity to use gathered data for preventive measures.

### Communicative Validation

In the second round of interviews, a communicative validation of the LAMH model was performed to evaluate whether the model matched the perceptions and experiences of the participants and refined it. When the LAMH model was presented first without any introduction, all participants reported that they considered the model a clear representation and felt that they recognized the displayed differences in adoption of eMental health from their experiences in daily clinical practice. When asked to which level they would classify themselves, all participants were able to categorize themselves to 1 level or in between 2 consecutive levels. This self-classification rarely diverged from the classification a priori made by the interviewer (ie, never more than 1 level higher or lower), based on the interview data. In the few cases (N=3) where there was a small divergence between interviewer classification and self-classification, the self-classification was always in the direction of higher acceptance of technology—that is, toward a higher level in the model. In general, participants agreed with the factors in the model, in particular those that were shared by all groups or those related to their own level:

I think these levels reflect the current situation very aptly, the different phases that you can be in and the barriers you experience.

P12

On the basis of the results, a few relatively minor adaptations were made to the LAMH model; some elements were added or removed, and some of the elements were rephrased to better match participants’ experiences. With respect to the layout of the model, some of the elements were reordered and lines were added to make it easier to understand. Figure 1 shows the final LAMH model.

## Discussion

### Principal Findings

Technology-mediated therapeutic tools have great potential in supporting the clinical practice of psychologists, and there is a substantial and growing evidence base in support of the efficacy of eMental health. However, at present, such tools are being underused in clinical practice, with only a minority of mental health care professionals and organizations employing a strategy to implement and use these new technologies. The discrepancy between the promise of eMental health tools and the documented reality of their use raises the following questions: why have these tools not been embraced more fully, what are the underlying barriers that hinder the adoption of eMental health tools, what are the perceived drivers that would help increase the acceptance and use of such tools, and How can these factors be structured in a way that improves systematic understanding? This study was aimed to elucidate these questions. In this context, research on technology acceptance provided a useful lens through which the drivers and barriers that clinical psychologists report could be analyzed and structured. On the basis of this, the authors developed a conceptual model for understanding the adoption of eMental health tools.

To arrive at these insights, this study utilized a qualitative descriptive approach consisting of in-depth interviews with clinical psychologists. The results indicated, first, that all participants consider a minimum amount of face-to-face contact vital to the quality of their treatment and stress the importance of basing their choice of eMental health tool on the needs and capabilities of the specific client. A major driver to the adoption of eMental health for psychologists is the belief and personal experience that eMental health can be beneficial for them or their practice, as it increases intrinsic motivation to use eMental health. Perceived benefits consist of the improvement of the therapeutic relationship, acceleration of the treatment process, increased satisfaction of client needs, personal benefits for therapists, and new treatment possibilities. Barriers that are most frequently reported are as follows: lack of knowledge and experience, increased demands due to increased accessibility, and technological issues. Furthermore, several contextual factors in daily clinical practice emerged as impeding factors to the adoption of eMental health, most notably lack of time and resources, feeling forced to use eMental health, and low visibility and awareness of eMental health.

Many of our findings are in line with results from previous research on the adoption of eMental health. Earlier studies also found lack of knowledge and experience, limited time and resources, and technological issues to be the most important impeding factors (eg, [9,14,37]. A barrier not highlighted in previous research pertains to the perceived increase of work demands caused by increased accessibility and a sense of moral responsibility beyond working hours implicated by the use of eMental health tools. However, such increased expectations and responsibilities associated with the use of new communication technologies have been identified outside the realm of mental health care, for instance in work focusing on social awareness systems within domestic settings [49,50]. In contrast to earlier work reporting that psychologists are influenced by social pressure from colleagues to reject eMental health [14,19], this study found that the general attitude in the workplace seems to be rather more disinterested than judgmental, as the topic of eMental health is rarely being discussed. In other words, in the context of day-to-day work pressures, exploring eMental health tools has a relatively low priority and visibility.

Compared with prior work in this area, this study puts a greater emphasis on the identification of drivers to the adoption of eMental health. Practitioners’ belief in the beneficial outcomes of eMental health is a key driver of its adoption. Such a benefit-driven approach is not uncommon in the motivated acceptance and use of new communication technologies and resonates with earlier studies pointing to the importance of users perceiving benefits of an innovation as a precondition of use [39,40]. The results suggest that clinical psychologists perceive the acceleration of the treatment process as a primary advantage. One plausible factor causing this acceleration, suggested by various participants in this study, is that mediated contact in-between regular sessions intensifies the treatment and affords a more intimate and personal therapeutic relationship. This explanation is in line with research suggesting that higher session frequencies are related to faster clinically significant gains in recovering from psychological distress [51].

In addition, although previous studies on therapists’ attitudes regarding eMental health have mainly resulted in an undifferentiated list of barriers and drivers, this study is the first to recognize a systematic relationship between the extent to which psychologists have adopted eMental health and the particular drivers and barriers they experience. On the basis of this insight, the LAMH model was developed that incorporates these differences. The model distinguishes 5 levels of adoption of eMental health and the corresponding drivers and barriers perceived by psychologists, determined based on several characteristics regarding their attitude and use of eHealth. At the heart of the LAMH model is the proposition that the willingness and ability of clinical psychologists to use technology-mediated therapeutic tools within their clinical context is contingent on the extent to which specific informational, motivational, technical, and organizational barriers at different stages are overcome and that specific drivers are present that will motivate and support the adoption process. Each level of adoption has a set of associated drivers, barriers, and requirements for change specific to that level. Although some barriers may be detrimental to adoption across all levels of the LAMH model—for example critical functionality limitations or severe usability problems associated with the eMental health tools—the model nevertheless assumes that lower level drivers and barriers will not be as relevant to higher levels of adoption, and vice versa. For example, at the levels 4 (active use) or 5 (innovative use) of the LAMH model, one would not expect that professionals experience a lack of knowledge about eMental health—a barrier that is typically found at lower adoption levels. Similarly, at level 1 (no use), one would not expect professionals to entertain a positive future vision on the role of eHealth in mental health care—a driver that does characterize professionals at level 5 (innovative use). The model also reveals that psychologists having lower levels of adoption experience relatively more barriers than drivers, whereas this balance shifts to the opposite for higher levels, which in turn could result in an increase of intrinsic motivation to continue or even expand the use of eMental health tools.

At any point in time, the LAMH model could be applied to determine the momentary level of adoption of eMental health by an individual mental health care professional, for example, to gauge individuals’ readiness to accept new technology-mediated therapeutic tools or to support organizational decision making when choosing a strategy to implement these innovative technologies. From the LAMH model, it can be inferred that interventions to increase adoption should be tailored to the practitioners’ individual level of adoption of eMental health. Even stronger, the model can be practically applied by informing how this level can be influenced on a much more specific level than was previously possible by enabling the development of interventions that are tailored to one’s level of adoption, hence targeting the specific barriers and drivers that are experienced. In addition, the model can be used to facilitate discussions among mental health care professionals about eMental health by forming a recognizable starting point and providing a shared language.

However, the model can also be conceptualized as a dynamic description of the process and stages of change. By distinguishing different levels of adoption and corresponding relevant drivers and barriers, the LAMH model enables examining the transitions between the levels, how these factors vary for different transitions, and their influence relative to each other. Besides relating specific drivers, barriers, and requirements for change to one’s level of adoption, the results can also be structured into various dimensions that are at play in the adoption of eMental health such as compatibility with the current way of working, personal innovativeness, usability of technology, and organizational support. Moreover, the results suggest that these dimensions play a different role for different levels of adoption. Clustering the themes in this way thus provides another view on the factors that influence this process. The gained insights could be further expanded by investigating how the levels of adoption of eMental health defined in this study relate to the existing models describing processes of behavior change and implementation that use similar dimensions such as the DIT [27] and the TAM [30].

Following the construction of the LAMH model, a questionnaire is currently under development that will allow fast and easy assessment of the level of adoption of eMental health by mental health care professionals. Such an instrument will be a powerful tool in conducting research on larger samples of clinical professionals and relating levels of adoption to, among others, specific therapeutic approaches, technological innovations, and organizational contexts.

### Limitations

A study of this kind has a number of limitations. Even though the participants in this study were carefully selected to comprise a representative sample of clinical psychologists, a limitation of this study is that it consisted of in-depth qualitative research with a small sample compared with most of the conducted studies with a quantitative approach. Conducting studies with larger sample sizes (enabled by the use of the assessment instrument that is being developed) will allow for further validation of the LAMH model. Moreover, although many of our insights are likely to hold true for the larger population of mental health care professionals, there may be subtle differences in experienced drivers and barriers across different mental health care occupations, which include psychotherapists, clinical psychologists, psychiatrists, and psychiatric nurses. This will be a subject for future investigations. Finally, although during the communicative validation phase of the study most participants agreed to their LAMH classification, few small divergences could be observed. These divergences pointed in the direction of a higher technology acceptance level, which is suggestive of a potential small social desirability bias; that is, it may be more desirable to come across as technology-savvy and open to technological innovations in mental health care. Although only a minor potential effect in our study, it is an issue that researchers need to be aware of when investigating technology acceptance among highly trained professionals.

### Conclusions

By studying the adoption of eMental health tools by clinical psychologists, this study contributes to their effective use, supporting the availability of timely, high-quality, well-integrated mental health care. The mediated nature of these tools has many well-documented advantages that serve this purpose, which makes the low utilization of eMental health a challenge that urgently needs to be addressed and resolved. This study addresses this need as it aimed to produce a more comprehensive and systematic understanding of this problem space. This goal is achieved by clarifying both barriers and drivers, several of which are new to the literature. In addition, the authors identified a relationship between the actual level of adoption and level-specific drivers and barriers and subsequently structured the obtained findings through the construction of the LAMH model. In this way, the authors hope to advance the existing insights into the specific drivers and barriers relevant for specific groups of mental health care professionals. In turn, these insights will inform processes of technology development, interface design, professional training, clinical use, and organizational embedding of eMental health tools that enable reaching the full potential of eMental health. Eventually, this is likely to bring significant improvements to the quality and efficiency of mental health care practice, from which both professionals and clients will benefit.

## Abbreviations

DIT: diffusion of innovations theory
eMental health: electronic mental health
LAMH: Levels of Adoption of eMental Health
TAM: technology acceptance model
